# Support for the Time-Varying Drift Rate Model of Perceptual Discrimination in Dynamic and Static Noise Using Bayesian Model-Fitting Methodology

**DOI:** 10.3390/e26080642

**Published:** 2024-07-28

**Authors:** Jordan Deakin, Andrew Schofield, Dietmar Heinke

**Affiliations:** 1 School of Psychology, University of Birmingham, Edgbaston, Birmingham B15 2TT, UK; jordan.deakin@uni-hamburg.de; 2 Faculty of Psychology and Human Movement Science, General Psychology, Universität Hamburg, Von-Melle-Park 11, 20146 Hamburg, Germany; 3 School of Psychology, Aston University, Birmingham B4 7ET, UK; a.schofield@aston.ac.uk

**Keywords:** selective influence, Bayesian cognitive modelling, perceptual decision making, perceptual integration

## Abstract

The drift-diffusion model (DDM) is a common approach to understanding human decision making. It considers decision making as accumulation of evidence about visual stimuli until sufficient evidence is reached to make a decision (decision boundary). Recently, Smith and colleagues proposed an extension of DDM, the time-varying DDM (TV-DDM). Here, the standard simplification that evidence accumulation operates on a fully formed representation of perceptual information is replaced with a perceptual integration stage modulating evidence accumulation. They suggested that this model particularly captures decision making regarding stimuli with dynamic noise. We tested this new model in two studies by using Bayesian parameter estimation and model comparison with marginal likelihoods. The first study replicated Smith and colleagues’ findings by utilizing the classical random-dot kinomatogram (RDK) task, which requires judging the motion direction of randomly moving dots (motion discrimination task). In the second study, we used a novel type of stimulus designed to be like RDKs but with randomized hue of stationary dots (color discrimination task). This study also found TV-DDM to be superior, suggesting that perceptual integration is also relevant for static noise possibly where integration over space is required. We also found support for within-trial changes in decision boundaries (“collapsing boundaries”). Interestingly, and in contrast to most studies, the boundaries increased with increasing task difficulty (amount of noise). Future studies will need to test this finding in a formal model.

## 1. Introduction

A part of Karl Friston’s massive and highly influential body of work is concerned with understanding human behavior by employing mathematical forms of theories. In Karl’s work, the theoretical framework is mainly the predictive coding theory mathematically framed in terms of the Free Energy principle [1,2,3,4,5]. Here, we also take a computational approach to understanding human behavior and focus on decision making in a noisy visual environment (e.g., in which direction an object is moving or what the name of an object is). The mathematical framework in this paper is the drift-diffusion model (DDM). The model assumes that decisions are made by sequentially sampling evidence from a stimulus and accumulating that evidence over time until a criterial amount is reached. The assumed stochasticity in the accumulation of evidence allows the DDM to capture the typical variation in both the speed (response time distribution) and accuracy of many decision processes. Consequently, the model has been applied to various aspects of cognition, including the processing of multiple object scenes in visual search tasks [6], word recognition in lexical decision tasks [7] and the discrimination of motion direction in fields of moving dots (random-dot kinematograms RDKs [8,9,10,11,12,13,14]). 

**Figure 1:**
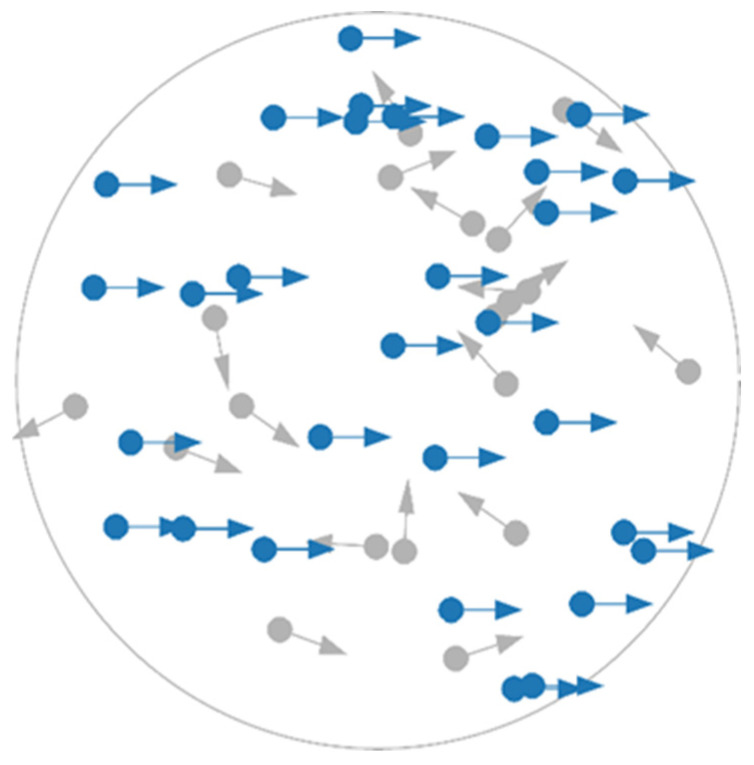
Example random-dot kinematogram (RDK) stimulus. In the typical RDK task, participants are presented with a field of randomly moving dots and asked to judge the motion direction of the coherently moving (blue) dots. The noise present (i.e., difficulty) is manipulated by varying the percentage of coherently moving dots relative to randomly moving (grey) dots. Here, arrows indicate direction of movement and colors are for illustrative purposes only.

Not only is the DDM behaviorally valid, but its assumptions are also supported by neurophysiological studies [15,16] examining neural firing rates in the lateral intraparietal area (LIP) when subjects view RDK stimuli, a frequently used stimulus in perceptual decision-making experiments (e.g., [12], see Figure 1 for an illustration). However, a potential shortcoming of the DDM is that it assumes the evidence accumulation process operates on a fully formed representation of perceptual information, available immediately after a brief encoding stage. In other words, it presumes evidence accumulation begins only once stimuli are fully encoded, thereby oversimplifying the integration of perceptual information. This assumption overlooks the possibility that integration may be stimulus-dependent and could simultaneously influence the decision-making process.

For example, forming a representation of luminous disks is very quick (around 100 ms), whereas the formation for RDKs’ motion direction is considerably longer at around 400 ms [17,18]. Considering this long formation period, it seems important for a model to acknowledge that a meaningful proportion of the evidence accumulation begins before the representation formation is completed. Recently, Smith and colleagues proposed an extension of DDM to rectify this shortcoming [19]. Their model, which here we refer to as time-varying DDM (TV-DDM), introduces a mechanism of perceptual integration by which the representation of perceptual information is formed over time during the decision process and modulates the speed (drift rate) of evidence accumulation. Indeed, a series of studies by Smith and colleagues has shown that a model incorporating time-varying drift rates well accounts for responses to stimuli (letters, bars, and grating patches) embedded in dynamic noise compared to the standard DDM [20,21]. It is also worth noting that Heinke and colleagues have introduced a similar type of model for reaching movements in which movements are assumed to be executed in parallel to the selection of the movement target (see [22,23] for details). Consequently, the movement control of the reaches is also affected by the target selection process analogous to how the formation of perceptual representation affects the decision process in TV-DDM. The assumptions of the TV-DDM not only incorporate the effect of perceptual integration on the decision process, but Smith and Lilburn [19] also demonstrated that the DDM’s disregard for this assumption can be particularly problematic. Specifically, when applied to the RDK task in which encoding is extended over time, the DDM’s assumption of an abrupt onset of perceptual evidence can lead to clear violations of “selective influence” and, in turn, false inferences [19,24].

The concept of “selective influence” goes back to Sternberg’s [25] additive factor method (see also [26] for a summary) and is set within a classical analysis of variance (ANOVA) framework of reaction time analysis. The assumption is that if an experimental factor is processed only by a specific corresponding cognitive process, then manipulation of this factor should only affect the processing duration within this cognitive process while other cognitive processes are unaffected. Importantly if two or more factors are varied in an experiment and these factors each tap into a separate process, ANOVA should only show an additive effect of these factors and no significant interaction. However, if factors share cognitive processes, significant interactions should be found. In model-based approaches, the concept of selective influence applies to parameters, which are assumed to represent cognitive constructs. Indeed, a key benefit of computational models is their ability to decompose overt behavior into latent cognitive constructs via their parameters. Hence, experimental manipulations designed to influence a given cognitive process should selectively influence parameter values that represent said process, while leaving other parameters unchanged (see [27,28] for a more formal treatment). Importantly, if a model is unable to accurately discriminate between different cognitive constructs through its parameters, then this benefit is undermined. At this point, it may be worth mentioning that this framework is very different from the currently very popular approach with deep neural networks where no such link with theoretical constructs exist, leading to a black box (see [29] for a detailed discussion).

The DDM possesses three parameters with clear predictions for experimental manipulations of selective influence: speed of accumulation of evidence (drift rate); the amount of evidence needed to generate a response (response boundary); and the necessary time for encoding perceptual information plus execution time for the motor response (non-decision time). Under a strict selective influence assumption, the discriminability of stimuli should only affect the drift rate, instructing participants to respond either quickly or accurately should affect only the response boundary (less (more) evidence required for fast (accurate) decisions) and response handicapping should only affect non-decision time. However, there is now a good amount of evidence for violations of selective influence. 

For instance, studies exploring selective influence violations have focused heavily on the effect of speed–accuracy instructions and have reported that these instructions affect not only response boundary but also estimates of drift rate [30,31,32] and non-decision time [33,34,35,36,37]. For example, Voss et al. [37] used a color discrimination task in which speed–accuracy instructions were found to influence both response boundary and non-decision time but not drift rate. Starns et al. [38] reported effects of speed instructions on all three parameters in an item recognition task. Rae et al. [32] also reported that emphasizing speed results in decreased estimates of drift rate, and reanalysis of these data by Ratcliff and McKoon [39] found additional decreases in non-decision time also. A similar finding was reported by Dambacher and Hübner [40] using a DDM-based model of the flanker task, where increased time pressure led to decreased estimates of response boundaries, non-decision time and drift rates for both early response selection and stimulus selection. The strongest evidence for violation of selective influence through manipulations of response caution comes from an RDK study by Dutilh et al. [24]. The authors manipulated difficulty (two noise levels), speed–accuracy trade-off (speeded response vs. accurate response) and response bias (proportion of left/right stimuli). A total of 17 modelling teams were invited to fit a model of their choice to a subset of the data and were asked to infer from these fits which experimental manipulations had been made, with most researchers using some variant of the diffusion model. Overall, the modelling results indicate that the DDMs tend to conflate manipulations of response caution with changes in non-decision time, where estimates for non-decision time are higher when participants are instructed to respond more cautiously. Importantly, Smith and Lilburn [19] replicated these findings but also demonstrated that this violation of selective influence is largely reduced by TV-DDM, underlining the importance of a perceptual integration stage.

It is important to note that not only have these speed–accuracy instructions been shown to influence estimates of response boundary, but there is also evidence that this manipulation leads to participants adopting response boundaries that “collapse” over time (e.g., [41]). Here, the assumption is that participants utilize time-dependent response boundaries to optimize their performance ([41,42,43] for examples of formal models). Typically, it is assumed that these within-trial changes in response boundaries occur when costs are associated, or deadlines are employed (see [41] for an example). However, a theoretical study by Malhotra et al. [44] showed that a mix of easy and difficult decisions can also lead to the same effect. Similarly, it has been proposed that adopting a collapsing boundary is an optimal strategy when decision evidence is unreliable [45], or when decisions are difficult (i.e., in difficult trials, subjects may reduce the criterial amount of evidence needed near the end of the trial in order to reserve cognitive resources, so that the next trial can be initiated [46]). On the other hand, a theoretical study by Boehm et al. [47] demonstrated that for most levels of difficulty, static response boundaries are robust and only at extreme levels are collapsing boundaries more optimal. Empirical support for collapsing boundaries comes from a study by Lin et al. [6] using the EZ2 variant of a DDM with data from a visual search task where participants are tasked with finding a designated target among non-targets. They found that the response boundary increased with increasing number of objects on the screen (i.e., the difficulty of the search task). Together, these findings imply a relationship between task difficulty and boundary, an effect which may manifest itself in apparent violations of selective influence in fits of the DDM.

The present study aims to follow up these few studies and examine violations of selective influence by manipulating task difficulty with a standard RDK stimulus (Study 1). To realize the most complete test, we let all main parameters vary freely across all noise levels in both models, DDM and TV-DDM. From a strict viewpoint, both models should be able to explain the data with only changes in drift rate. However, the lack of integration of perceptual information in a standard DDM suggests that non-decision time may not be constant across the noise levels as pointed out by Smith and colleagues. In contrast, TV-DDM should be able to mitigate this problem. Noteworthy, Dutilh et al. [24] did not report a violation of selective influence for this experimental manipulation. However, this may have been due to the limited number of noise levels. Here, we will test a wider range of noise levels (10%, 40%, 70%, and 80%). Finally, Malhotra et al.’s [44] study predicts that we could find within-trial changes in decision boundaries (i.e., different boundaries for difference noise levels) as the study mixes up easy and hard conditions. In a second study, we introduced a novel type of noise manipulation where we maintained the spatial structure of the RDK (i.e., randomly placed dots in an aperture). However, rather than randomizing the motion direction, we randomized the hue of the noise dots, used a certain hue (cyan or red) for signal dots (rather than left or right moving dots), and varied the proportion of signal dots analogous to the noise manipulation in RDKs of coherently moving dots. Hence, participants were asked to perform a color discrimination task while static noise was manipulated. We termed these novel stimuli random dot patterns (RDPs). These stimuli allowed us to test a potential confounding factor in Smith et al. [21] which showed perceptual integration is pertinent in dynamic noise (see above). However, their stimuli with static noise manipulation requires only little integration of spatial information while RDKs require a good amount of such integration. Hence, it is conceivable that the support for TV-DDM may have been also due to this time-consuming spatial integration and not only the dynamic noise. Hence, if our observation is correct, we expect TV-DDM to also be superior for the RDPs as they also require spatial integration, like RDKs. Finally, it is important to note that we fit the models using Bayesian parameter estimation to test the selective influence assumption, and for the model comparison we used the ratio between marginal likelihoods (Bayes factor). This Bayesian methodology allowed us to consider prior knowledge about parameter estimations from Smith and Lilburn’s [19] maximum likelihood estimate for both models.

### 1.1. Model Descriptions

#### 1.1.1. DDM

DDM’s evidence accumulation process is described by a Wiener process with drift rate *v*, which determines the speed and accuracy of the evidence accumulation process. The change in evidence (*x*) at any time point is described by:

(1) $$\Delta x_{t}=vdt+\sigma \Delta W_{t}$$

where *dt* is a small timestep, *σ* is the diffusion coefficient which controls the noise in the process, and *W* represents the Wiener process, which adds normally distributed noise to the accumulation. 

**Figure 2:**
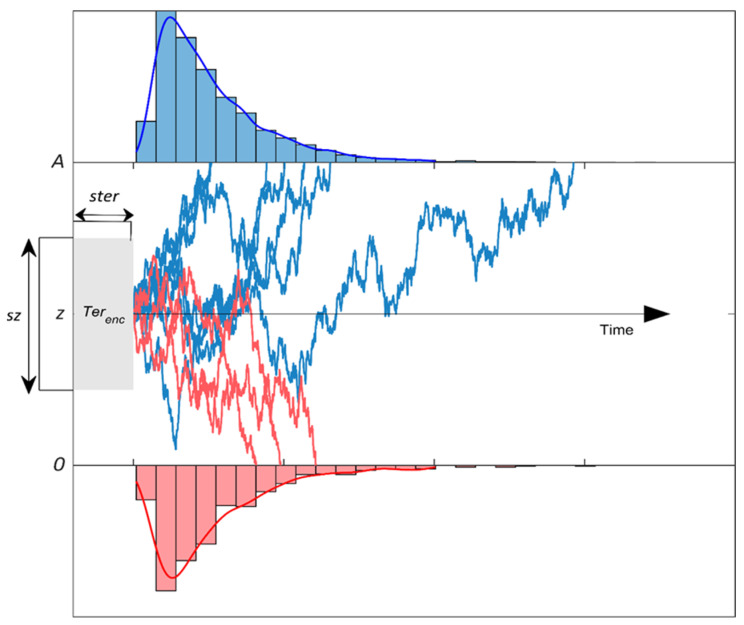
Schematic diagram of the drift-diffusion model. Blue (red) traces represent the noisy evidence accumulation process for correct (incorrect) trials and histograms show the resulting reaction-time distributions. If the process hits the upper (lower) boundary, a correct (error) response is made.

Figure 2 shows the resulting noisy accumulation of decision evidence. The diffusion process begins at start point *z* (usually *A*/2 assuming no bias) and accumulates evidence towards one of two response boundaries. If the diffusion process hits the upper boundary *A*, the correct response is given, whereas if the lower boundary is hit, an error response is given. The DDM assumes total RT is a sum of decision time (the time taken for the process to hit a boundary) and non-decision time *Ter*. Non-decision time encapsulates both the time required for stimulus encoding (*Ter_enc_* in Figure 2) and the time taken to execute the motor response following a decision. The DDM also assumes some variability in start point (*sz*), non-decision time (*ster*), and drift rate (*sv*), which allow the model to account for a wider range of data patterns, particularly those regarding the relationship between RT and accuracy [48,49]. Both *sz* and *ster* are assumed to be drawn from uniform distributions, while drift-rate variability (*sv*) is drawn from a normal distribution. Here, we fit the DDM with 6 free parameters, namely response boundary (*A*), non-decision time (*ter*), drift rate (***v***), and the three between-trial variability parameters (variability in start point (*sz*), drift rate (*sv*), and non-decision time (*ster*)).

#### 1.1.2. TV-DDM

**Figure 3:**
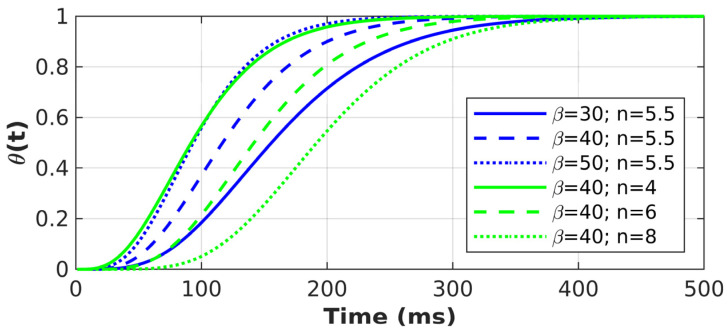
Illustration of the gamma function for a range of parameter settings.

For TV-DDM, Smith and Lilburn [19] extended the Wiener process equation:

(2) $$dx_{t}=v\;\theta \left(t\right)dt+\sqrt{\sigma^{2}\theta \left(t\right)+\sigma_{2}^{2}}\Delta W_{t}$$

by adding an additional variance, σ_2_, and θ(t), a “growth-rate function”. σ_2_ aims to capture premature sampling prior to stimulus onset. The evidence growth term aims to reflect integration of perceptual information with dynamic noise described through an incomplete gamma function (see Figure 3 for an illustration):

(3) $$\theta \left(t\right)=\frac{1}{\Gamma (n)}\;\int_{0}^{\beta t}e^{-s}s^{n-1}ds$$

with *β* (evidence growth rate) and *n* (evidence growth shape) as parameters and *Γ(n)* as the gamma function. The gamma function can be interpreted as describing n-stages of linear filters, creating a representation of the perceptual information starting at no representation (0%) and leading to full representation (100%). 

Figure 3 illustrates the influence of the parameters on this build-up. Note that the chosen values are in the range of the model-fitting results. An intuitive description of how the parameters affect the function can be that both parameters influence the speed of the build-up, but n has a particularly strong influence on the onset of this build-up, consistent with representing the number filtering stages. In summary, TV-DDM has the following parameters: response boundary (*A*), non-decision time (*ter*), drift rate (v), the evidence growth rate (*β*), and shape (*n*) parameters, premature sampling noise (*σ2*), and the between-trial variability in drift rate parameter (*sv*). Hence, TV-DDM has one more free parameter than DDM.

## 2. Materials and Methods

### 2.1. Modelling Procedures

#### 2.1.1. Bayesian Parameter Estimation and a Posteriori Estimates (EAP)

Model simulations, parameter estimation, and model comparison were all performed in MATLAB R2021b [50]. Parameters were estimated via Differential Evolution Markov Chain Monte Carlo (DE-MCMC). The sampling process in DE can take on different aims by the way new samples (proposals) are generated. These different transition rules are either better suited to find the best-fitting parameters or to determine posterior distributions (see [51,52] for examples). Here, we employed two steps based on two different transition rules. The first transition rule, the current-to-best algorithm, aims to find the best-fitting parameters, whose final samples form the starting values for the second step, where the DE-MCMC transition rule generates posterior distributions (see [53] for both transition rules). For the first step, we used 1000 iterations (generations) and 30 chains, while for the second step, we used 30 chains and 3000 iterations, 500 of which were considered part of the burn-in period and therefore discarded. This meant that for each parameter, we obtained 90,000 posterior samples. We fit the models to each participant and noise level separately.

The prior distributions were truncated normal distributions (see Table 1) and their parameters were informed by Smith and Lilburn’s [19] results, presented in their paper in Table 4 for DDM and Table 7 for TV-DDM. In fact, we chose similar values for the mean value of the priors with three exceptions. For the diffusion rate, we chose a midrange value for the mean, as it is not exactly clear how their difficulty manipulation compares to ours. Second, we noted that our participants were generally slower and more accurate than Dutilh et al.’s [24]. This may have been due to the fact that our study was completed online, and the presentation time of the stimuli was unlimited (while Dutilh et al.’s presentation time was limited to 3s). Therefore, we chose a value at the high end. Third, we chose higher values for the between-trial variability as, again, the nature of the online study may have led to more variability in their responses.

To approximate the likelihood function for both models, we followed Turner and Sederberg’s [54] method and utilized a kernel density estimator (KDE) to determine likelihood values for a given parameter setting. This way, both models are put on an equal footing. As in Narbutas et al. [55], we utilize a KDE method termed on-line KDE (oKDE [56]), a probability density approximation method which estimates the shape of a density function using a combination of Gaussian kernel distributions. Compared to the traditional KDE proposed by Silverman [57], the oKDE method is more flexible, since it optimizes the number of kernels and their widths. Hence, oKDE leads to a more efficient and more adaptable KDE than the standard KDE method ([55,56] for detailed discussion). This allowed us to efficiently estimate the density functions of each model, under which the data could be evaluated to compute likelihood values. Here, we created the KDE from 1000 simulated trials of a model. Both models were simulated in seconds using Euler’s method, with a diffusion coefficient of 0.1 and an integration constant of 1ms. Since the estimation of likelihoods via simulation can be highly variable, we also added a “purification step” with 30% probability into the DE-MCMC algorithm [58]. This step ensured that the chains did not get ‘stuck’ in areas of artificially high likelihood, leading to better mixing amongst the chains. To assess convergence, we calculated split $\hat{R}$ [59], a diagnostic statistic which evaluates convergence by comparing within-chain variance to between-chain variance, with $\hat{R}$ <1.1 being optimal. For all results presented, $\hat{R}$ was well below the criterial value of 1.1. The posterior distributions were estimated for each participant and each noise level separately. To summarize the posterior distributions, we calculated the expected a posteriori (EAP) estimates, taking the mean of each marginal posterior for each participant and averaging the results.

#### 2.1.2. Model Comparison

The models were compared using the ratio of marginal likelihoods (Bayes Factor, [60]). The calculation of the integral version of the marginal likelihood is computationally expensive as it requires the solving of a multi-dimensional integral across the whole parameter space (albeit limited by the priors). Here, we approximate this integral using Thermodynamic Integration for DE-MCMC (TIDE; [61]). For our purpose, this method is convenient as it requires only a simple extension of DE-MCMC. We used 10,000 generations with 30 chains (temperatures), which were initialized through the EAPs and omitted the first 1500 samples as a burn-in period when estimating the marginal likelihood. Finally, to assess the strength of the evidence for the two models, we used the interpretation proposed by Jeffreys [62]. This interpretation of the Bayes factor (BF) distinguishes six categories: decisive evidence for Model 1 (BF > 100) or for Model 2 (BF < 0.01), very strong evidence for Model 1 (BF: 30–100) or for Model 2 (BF: 1/100–1/30), strong evidence for Model 1 (BF: 30–100) or for Model 2 (BF: 1/100–1/30), moderate evidence for Model 1 (BF: 10–30) or for Model 2 (BF: 1/10–1/30), anecdotal evidence for Model 1 (BF: 3–10) or for Model 2 (BF: 1/3–1/10), and no evidence (BF: 1).

Since the Bayes factor is often criticized as sensitive to the choice of priors (even though here, this sensitivity is part of the methodology, as the priors allow us to include results from Lilburn et al. [19]), we also calculated two standard criteria, the deviance information criterion (DIC) and the Bayesian predictive information criterion (BPIC).

#### 2.1.3. Data Analysis

Before any analysis, the data were cleaned. We removed participants whose accuracy was below chance (see individual study sections for details) and any RTs faster than 150 ms or slower than 3000 ms. To analyze average performance, median RTs and mean error rates for each participant were analyzed via ANOVAs or *t*-tests. We also present the results from distributional measures, namely the cumulative density functions (CDFs) and conditional accuracy functions (CAFs). To apply the distributional measures to the models, we generated 2000 trials per condition for each participant using their EAPs and averaged them to calculate group measures. CDFs illustrate the cumulative density functions of RTs by plotting the average RTs of each RT quantile on the x-axis and the corresponding quantile on the y-axis. We used 17 quantiles ranging from 10% to 90% in equal increments of 5%. Conditional accuracy functions (CAFs) plot the average RT quantiles for both incorrect and correct responses against accuracy for the corresponding quantile. The quantiles for the CAF in this paper were from 10% to 90% in equal increments of 20%. We chose coarser quantiles for the CAF compared to the RT measures (CDFs) to account for the fact that some conditions showed relatively small error rates.

To illustrate the uncertainty of these distributional measures due to the uncertainty of the parameter estimates (posterior distribution), we sampled 1000 parameter settings from the posterior of each participant, determined the distributional measures for each sample and each participant, averaged the distributional measure across participants for each sample and then calculated the 95% credible interval (we would like to thank the second reviewer for suggesting this method). The effects of noise on the EAP estimates were analyzed using a standard one-way ANOVA. To take into account the uncertainty of the EAP estimates in this analysis, we repeated the ANOVA with samples from the posterior distributions, and if there were sufficient significant ANOVAs, we decided that there was a statistically significant effect even when considering the uncertainty of the EAP estimates. Additional details of this method follow. For each participant and each noise level, we sampled *n* values (see below) from the posterior of a parameter, averaged them and conducted an ANOVA. We repeated this 1000 times and determined the proportion of significant effects. If there were more 95% significant effects, we considered this as a confirmation of the significant effect from the initial one-way ANOVA. In other words, if there was very little overlap between the posteriors as measured by the proportion of significant effects from the ANOVA, we interpreted this as a significant effect of noise on the parameter in question. We termed this new indicator *posterior overlap indicator*. In addition, to illustrate the uncertainty of the EAP estimates, we calculated the 95% credible interval from the posterior samples and displayed them in the EAP graphs (Figure 8). When we first tested this method, we set *n* to the same value as the number of posterior samples, as this setting is typically used to assess variability (bootstrap method). However, it turned out that this setting was too liberal, so we decided to sample only 10 values, as this provided a more conservative assessment of the overlap of the posterior distribution.

### 2.2. Experimental Method

Both experiments were hosted online using Gorilla Experiment Builder [63] and coded in JavaScript using the jsPsych 6.0.1 library for the creation of behavioral experiments [64]. Prior to the task, all participants completed a Virtual Chinrest Task [65], to ensure a viewing distance of approximately 60 cm (−/+5 cm). This, along with the resolution and dimensions of the participant’s screen were used to scale the diameter of the circular apertures used in both tasks to three degrees of visual angle. In both experiments, a trial began with 500 ms presentation of a white fixation cross. The subsequent RDK or RDP was presented until the participant responded. The response key was either ‘Z’ or ‘M’ for either movement direction or overall perceived color (red or cyan). Both experiments were in 2 by 4 design—four levels of noise and two response levels. Both factors were randomized.

#### 2.2.1. Motion Discrimination (RDK Task)

##### Participants

A total of 47 students (36 females, 11 males, age: 18–35 years, *M* = 20.2 years) from the University of Birmingham were recruited to take part in the study in exchange for course credit. Participants gave prior consent and were required to not suffer from migraine or epilepsy. One participant showed accuracy below chance level (48.2%) and was excluded, leaving 46 participants (35 females, 11 males, *M* = 20.2 years) in the final sample, with accuracy rates ranging from 70.3% to 100%.

##### Stimuli

The motion discrimination task employed circular RDKs created using the jsPsych RDK plugin [66]. Each RDK had a diameter subtending 3 degrees of visual angle, was presented on a black background and comprised 50 white dots, each with a diameter of 2-pixels, a speed of 1.5 pixels per frame and a lifetime of 30 frames (see Figure 1 for an illustration). If a dot hit the (invisible) aperture edge or exceeded its lifetime, it was regenerated on the opposite side

##### Procedure and Design

The task followed a 2 × 4 factorial repeated measures design with factors direction (left and right) and noise (10%, 40%, 70%, 80%). The noise factor determined the percentage of randomly moving dots in an RDK, while the direction factor determined the direction in which the remaining coherently moving dots travelled. Both factors were randomized across trials.

Each trial began with the central presentation of a white fixation cross for 500 ms before the RDK was presented. The RDK remained on screen until a response was made and participants were instructed to identify the coherent motion direction (either leftwards or rightwards) as quickly and as accurately as possible using the keyboard. All participants responded using the ‘Z’ and ‘M’ keys for left and right responses, respectively; since this provides an intuitive mapping of the leftwards response to the left hand and the rightwards response to the right hand, we decided not to counterbalance these keys across participants. The task was split into four blocks, each beginning with 16 practice trials followed by 32 experimental trials (four trials per combination of noise and direction). In practice trials, responses were followed by the presentation of the words ‘Correct’ or ‘Incorrect’ for 500 ms before the next trial began. In experimental trials, no feedback was given. At the end of each block, participants were required to take a minimum 60 s break before being prompted to continue when ready. By the end of the task, each participant had completed a total of 128 experimental trials (32 trials per noise level).

#### 2.2.2. Color Discrimination (RDP Task)

##### Participants

A new sample of 36 students (33 females, 2 males, 1 non-binary person, age: 18–22 years, *M* = 19.5 years) from the University of Birmingham were recruited to take part in the study in exchange for course credit. Participants gave prior consent and were required to not suffer from migraine or epilepsy. Participant accuracy ranged from 85.9% to 99.6%.

##### Stimuli

To create the RDPs, we modified the jsPsych-RDK plugin [66] and added an extra input of dot color while setting the speed of the dots to 0. All other properties (dot size, aperture size, background color) were identical to the RDK task. The color of the dots was set in HSL space, as it often seen as a good approximation of human color perception (e.g., [67]). 

**Figure 4:**
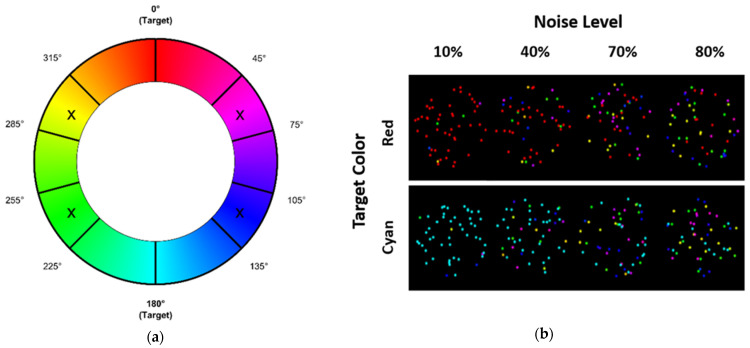
(**a**) Adjusted HSL color space. Areas marked ‘X’ are areas from which noise dot hues could be sampled, while target colors were true red or true cyan. (**b**) Example RDPs for each noise level and target color.

For all dots, saturation (S) was 100% and the luminance (L) was set to 50%, while the value of the hue was randomly varied to create an RDK-like manipulation (see Figure 4 for an illustration). In contrast to the RDK task, however, participants were asked to decide on the “coherent color” in the RDP. The signal colors were defined as true red (0°) and true cyan (180°). To ensure a difficulty similar to the RDK-stimuli, this hue was limited to four intervals 45–75°, 105–135°, 225–255°, and 285–315°, as these intervals make sure that the signal dots could be separated from the noise dots. Note that within each interval, 90 linearly spaced hues were sampled, creating 360 possible hues. To manipulate the noise in the stimuli, the percentage of noise dots was varied using the same noise levels as the RDK task (10%, 40%, 70%, and 80%). Figure 4a shows the HSL color space, with areas from which noise dot hues could be sampled marked ‘X’. Figure 4b shows example RDPs for each noise level and target color.

##### Procedure and Design

The task followed a 2 × 4 factorial repeated measures design with factors target color (red and cyan) and noise (10%, 40%, 70%, 80%). The trial-by-trial procedure for the color discrimination was identical to the motion discrimination task. Each trial began with the central presentation of a white fixation cross for 500 ms before the RDP was presented. The stimulus remained on screen until a response was made and participants were instructed to identify the coherent target color (either red or cyan) as quickly and as accurately as possible using the keyboard. All participants responded using the ‘Z’ and ‘M’ keys, yet the keys corresponding to red and cyan were counterbalanced across participants, with ‘Z’ mapped to ‘red’ for half of participants and vice versa for the other half.

The task was split into four blocks, each beginning with 16 practice trials followed by 32 experimental trials (four trials per combination of noise level and target color). In practice trials, responses were followed by the presentation of the words ‘Correct’ or ‘Incorrect’ for 500 ms before the next trial began. In experimental trials, no feedback was given. At the end of each block, participants were required to take a minimum 60 s break before being prompted to continue when ready. By the end of the task, each participant had completed a total of 256 experimental trials (64 trials per noise level). Note that the experiment was split into two sessions, with an equal number of trials and participants were asked to complete the second session with three days.

## 3. Results

### 3.1. Motion Discrimination (RDK Task)

**Figure 5:**
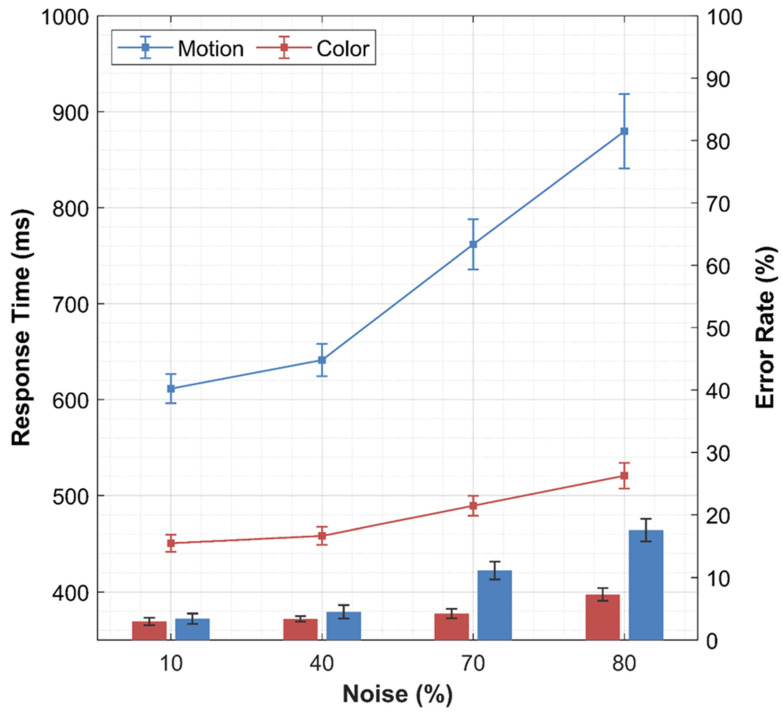
Average response time and error rate for the motion discrimination and color discrimination task for each level of noise.

Figure 5 shows the results of the average performance analysis. Prior to the analysis, outlier reaction times faster than 150 ms or slower than 3000 ms, accounting for 2% of the data, were removed. Median reaction times and mean arcsine transformed accuracy (both collapsed across motion direction) were aggregated across participants before being subjected to a one-way within-participants ANOVA with the factor noise (10%, 40%, 70%, 80%). The effect of noise on reaction times was significant (*F*(3,135) = 65.885, *p* < 0.001, $\eta_{p}^{2}$ = 0.594) with reaction times increasing with the level of noise. The effect of noise on accuracy was significant (*F*(3,135) = 69.653, *p* < 0.001, $\eta_{p}^{2}$ = 0.608), with accuracy decreasing with the level of noise. These results demonstrate that our noise manipulation was successful.

**Figure 6:**
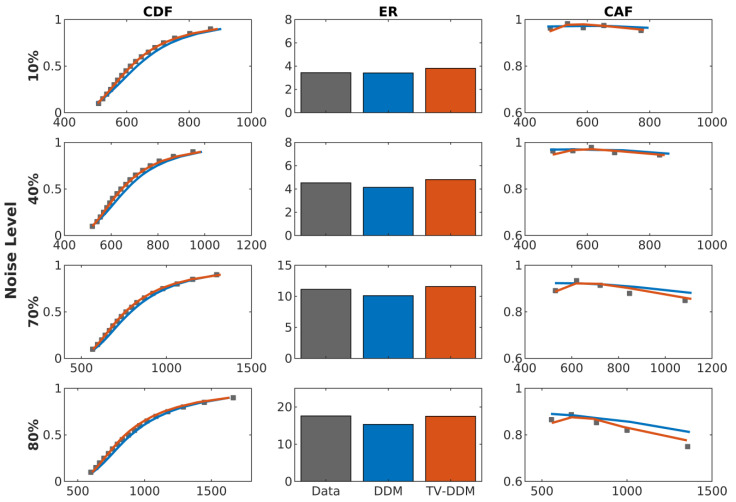
Cumulative density functions (CDF), average error rate (ER), and conditional accuracy functions (CAF) for the data and both models averaged over participants in the RDK task. The 95% credible interval is shown for these results are shown in Appendix A.

To assess the fit of the models to the data, we compared the fit of both models to the cumulative density functions (CDFs), conditional accuracy function (CAF), and overall error rate (ER). These results are shown in Figure 6. As the figure shows, both models fit the data relatively well; however, the fit of TV-DDM is notably better, particularly for lower levels of noise. Specifically, TV-DDM better predicts reaction times for intermediate quantiles at lower levels of noise and better predicts error rates at higher levels of noise. The largest discrepancy between TV-DDM and DDM, however, is in the CAF, where TV-DDM is superior at capturing slow errors at higher noise levels, as well as capturing the small increase in accuracy for the fastest responses.

**Figure 7:**
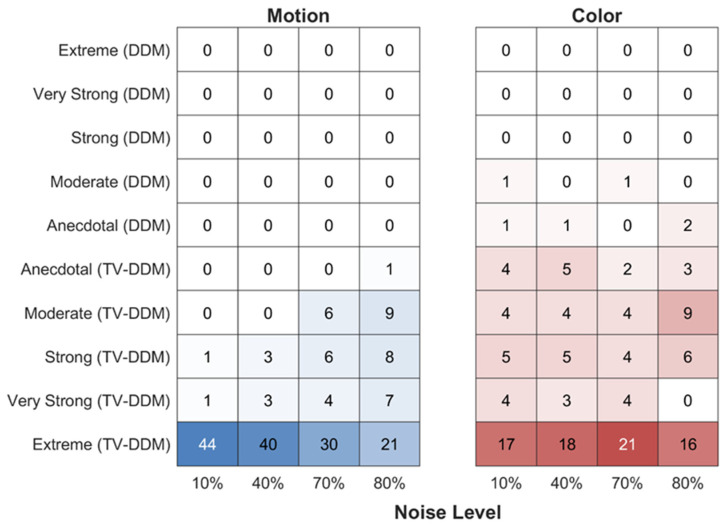
Strength and direction of model evidence for the motion discrimination (RDK) and color discrimination (RDP) tasks. Note that since we fit the models to each participant/noise level individually, the columns sum to the total number of participants (N = 46 for motion discrimination and N = 36 for color discrimination).

The results from the Bayesian model comparison for the motion discrimination task are presented in the left panel of Figure 7, which shows the number of participants who fall into each evidence category based on the strength and direction of evidence as specified by Jeffreys [62]. The results show that the data are overwhelmingly in support of the TV-DDM and are also in line with the fits to the distributional measures. Also note that DIC and BPIC also show the same results whereby BPIC indicate slightly less support for TV-DDM. This is expected, as BPIC considers the number of parameters more than DIC. As noise increases, the strength of support for TV-DDM decreases slightly. This may be since generally in the higher noise condition the results may vary more, plus that intermediate response times in the highest noise condition did not match the data (see CDF) as in the lower noise conditions. This may indicate that the gamma function may not capture the shape of the formation of perception at all noise levels. Overall, however, this difference was not sufficient to make the DDM the preferred model, suggesting the slightly increased complexity of the TV-DDM was justified.

**Figure 8:**
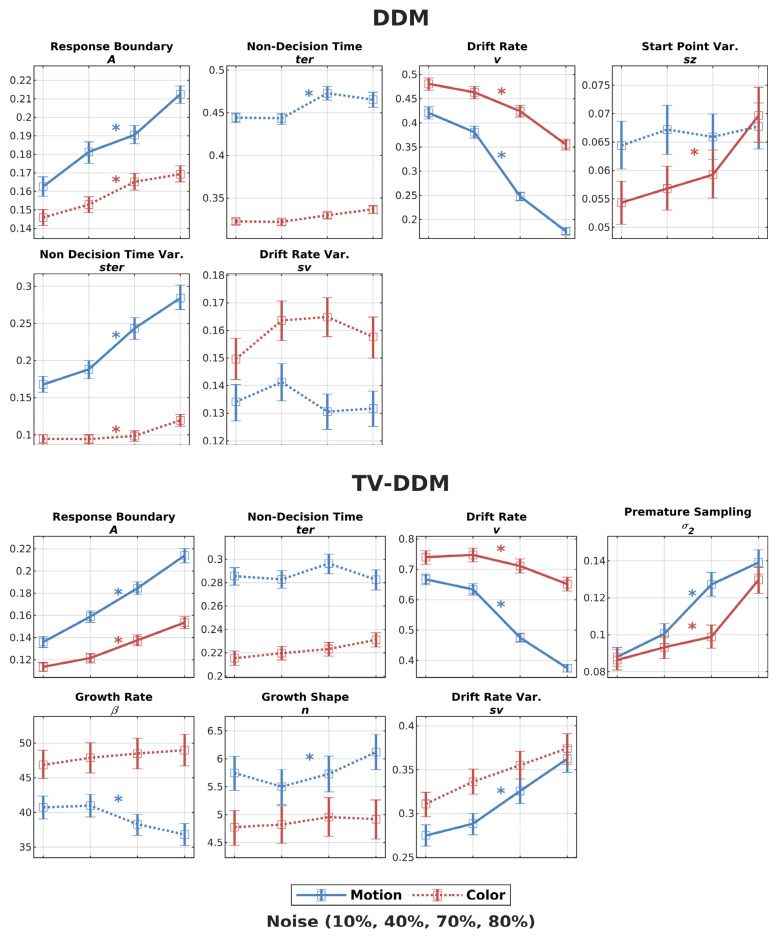
Average expected a posteriori (EAP) estimates for DDM and TV-DDM. The solid lines indicate a significant effect of noise in both measures, the one-way ANOVA and the posterior overlap indicator. The asterisk indicates a significant effect when considering the one-way ANOVA only. The error bars indicated the 95% credible interval to illustrate the uncertainty of EAP estimates (see method section).

The EAP estimates for each parameter were analyzed with a one-way ANOVA with noise as the within-participants factor and posterior overlapping indicator (see Appendix A for details). These EAP estimates for both models, together with the ANOVA results, are shown in Figure 8, where the estimates for the RDK task are shown in blue. As expected, both models show a clear decrease in drift rate as noise increases, consistent with the interpretation that drift rate accurately reflects task difficulty (discriminability of the stimuli). However, as it is clear from Figure 8, the noise manipulation also affects a number of other parameters in both models, violating the strict selective influence assumption. For the DDM, the decrease in drift rate across noise conditions is accompanied by an increase in the response boundary (*A*), and variability in non-decision time (*ster*). Similarly, for TV-DDM, response boundary also increases with increasing noise, and the variability in drift rate. We also calculated the point of time where the value of growth-rate function reaches 97%, as suggested in Smith and Lilburn [19]. This time can be related to the temporal integration times obtained by Watamaniuk and Sekuler’s [18] psychophysical experiments. For the average values of β and n, the temporal integration times fall between 271 ms and 314 ms. Finally, it is worth noting that we did not find support for a violation of selective influence in DDM’s non-decision time (*ter*) as we initially expected. Even though the one-way ANOVA of the EAP estimates was significant, the uncertainty of the EAP estimates was too high (posterior overlap: 0.24) to confirm the EAP effect. On the other hand, for TV-DDM, even the ANOVA was not significant, indicating that the effect of noise on non-decision time (*ter*) was more attenuated through perceptual integration in line with the one of the original motivations of TV-DDM.

### 3.2. Color Discrimination (RDP Task)

Figure 5 shows the results of the average performance analysis. Prior to the analysis, outlier reaction times faster than 150 ms or slower than 3000 ms, accounting for 0.23% of the data, were removed. Median reaction times and mean arcsine transformed accuracy (both collapsed across target color) were averaged across participants before being subjected to a one-way within-participants ANOVA with factor noise (10%, 40%, 70%, 80). The effect of noise on reaction times was significant (*F*(3,105) = 69.494, *p* < 0.001, $\eta_{p}^{2}$ = 0.665) with reaction times increasing with the level of noise (see Figure 3). The effect of noise on error rate was also significant (*F*(3,105) = 10.991, *p* < 0.001, $\eta_{p}^{2}$ = 0.239), with the error rate increasing with the level of noise. These results demonstrate that our noise manipulation was successful. Moreover, we compared the results with Study 1 through a two-way mixed ANOVA. We found that responses in the RDP task were significantly faster and more accurate than the RDK task, although the noise manipulation was less effective in terms of both reaction times and accuracy. Hence, the RDP task does not match the difficulty of RDK task. However, we believe that this does not affect the critical conclusions from the results, especially the modelling findings.

**Figure 9:**
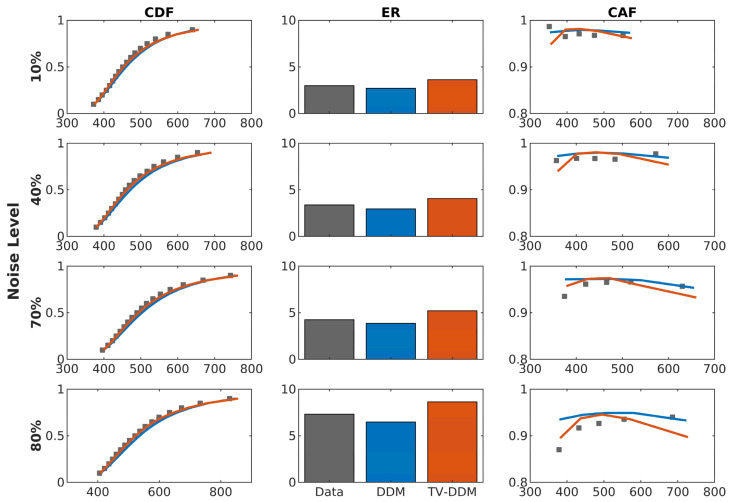
Cumulative density functions (CDF), average error rate (ER), and conditional accuracy functions (CAF) for the data and both models averaged over participants in the RDP task. The 95% credible intervals for these results are shown in Appendix A.

The distributional measures for the RDP task are presented in Figure 9. As with Study 1, the distributional measures (CAF, CDF) showed that TV-DDM is better at predicting increases in accuracy for the fastest response times. However, TV-DDM’s prediction of a decrease in accuracy for the slowest responses, which in Study 1 allowed it to model the data better than DDM, leads to a clear underprediction of accuracy for the slowest responses in the RDP task, whereas this is not the case for DDM. This observation is supported by the fact that TV-DDM overpredicts overall error rate, while the DDM’s tendency to overpredict accuracy for the fastest responses means that it slightly underpredicts overall error rate. These results indicate that the gamma function takes on a slightly different shape for color noise.

Despite these discrepancies, the model comparison (Figure 7) reveals very strong support for TV-DDM, similar to Study 1, albeit slightly less. Again, DIC and BPIC mirror these findings. This finding is not consistent with Smith and colleagues’ findings, which suggested that static noise does not require a perceptual integration stage.

The ANOVA analysis of the EAP estimates and the posterior overlapping indicator of both models exhibited a significant effect of noise on the response boundary (*A*), the drift rate (*v*), and premature sampling noise ($\sigma_{2}$), as with Study 1 (see Appendix A for details). A two-way mixed ANOVA with factors task (color and motion) and noise (10%, 40%, 70%, 80%) (only with EAP estimates; see Appendix A for details) showed that the response boundary was smaller and less affected by noise than in the RDK study. For the drift rate, the RDP task had a significantly larger drift rate than the RDK task as the response boundary was less affected by the noise level. Both effects support the behavioral analysis in that the RDP was an easier task. For the remaining important parameters, evidence growth rate, evidence growth shape, TV-DDM’s non-decision time, and DDM’s non-decision time, no effect of noise was found for the RDP task. For the average values of β and n, the temporal integration times fall around 202 ms.

## 4. Discussion

The aims of the paper were threefold: first, we aimed to replicate Smith and colleagues’ findings that their model, TV-DDM, is superior to the traditional DDM for modelling perceptual decisions about RDK stimuli; second, we aimed to test TV-DDM’s ability to model data patterns regarding the discriminability of stimuli without violating selective influence assumptions (amount of noise in RDKs across four levels 10%, 40%, 70%, and 80%) accurately and compared it to DDM in this respect; third, we tested TV-DDM with a novel type of stimuli (Study 2), a static color version of RDKs, here termed random dot patterns (RDPs). Again, this third aim explored the superiority of TV-DDM compared to DDM. Both studies fit and compare the two models using Bayesian parameter estimation and model comparison. We will begin this discussion with the more mundane aspects of our study, the psychophysical characteristic of our novel type of stimuli, and then turn to the more exciting topic of this paper, the modelling.

The behavioral analysis for RDP stimuli revealed that our manipulation of noise (proportion of noise dots) was successful as reaction time and error rate increased with increasing levels of noise. However, the effects were smaller in the RDP task compared to the RDK task, i.e., the overall performance (reaction times and error rate) was better and decreased less with increasing noise than in the RDK task. Hence, this color discrimination task is an overall easier task, which is also confirmed by the a posteriori estimates (EAP) of both models, e.g., response boundary, drift rate, etc. We think this is mainly due to the way the noise dots were chosen, i.e., intervals of hue fairly distinct to the signal dots. However, in brief pilot studies we found it difficult to hit a “sweet spot”, e.g., sampling noise dot hues in a similar way to the RDK (i.e., sampling from all 360 possible directions) made the task too difficult. However, there is also the possibility that color discrimination is easier than motion discrimination. Future research will need to explore this further. For the purpose of this paper, it is important to note that the noise manipulation was successful, which in turn allows us to contrast static noise with dynamic noise while important properties of both types of noise, especially the need to integrate across space to make the best decision, are maintained.

Now we turn to the modelling results of this paper. The model comparison using the Bayes factor revealed a strong win for TV-DDM for both studies despite TV-DDM having one parameter more than DDM (albeit less clear-cut for the RDP stimuli). The distributional measures for both studies revealed that TV-DDM is better equipped to predict the time course of accuracy than DDM. Specifically, the sharp increase in accuracy at short reaction times is well captured by TV-DDM, reflecting the effect of the growth-rate function. However, it is worth noting that the DDM with between-trial variability in start point (*sz*), drift rate (*sv*), and non-decision time (*ster*) (see [48,49,68,69]) should have been able to predict this pattern of fast and slow errors, but it seems the required flexibility in balancing the two types of errors was insufficient for this data. The finding that TV-DDM also wins for the RDP task is perhaps surprising as Smith and colleagues found that support for TV-DDM mainly from stimuli with dynamic noise (e.g., [17]). However, Ratcliff and Smith [70] also found that letters with static noise produce also support for TV-DDM, but the effects were small. Hence, a possible way of reconciling our strong support for TV-DDM for static noise with their findings is that both letters and our RDPs require some degree of integrating information across space, whereas RDPs require the consideration of a “larger” space than letters, explaining the difference in terms of support. Hence, it is not only dynamic noise which requires the perceptual integration stage, but also the integration of information across space. Of course, there is also the possibility that this is due to methodological differences. Future research will need to explore this hypothesis in more detail.

With respect to the test of selective influence, both studies revealed a mixed picture. To begin with, it is important to note that the effect of drift rate from both tasks revealed the expected noise dependency with drift rate decreasing with increasing noise. Also, TV-DDM was able to model the data without violating selective influence assumptions regarding non-decision time (non-decision time did not change across noise levels for both types of tasks). This was also true for DDM not supporting our expectation that lack of perceptual integration may lead to a violation of selective influence. However, our findings are in line with Dutilh et al.’s findings. Here we confirm them for a wider range of difficulties. It is conceivable that the other parameters (e.g., drift rate) are able to absorb the influence of perceptual integration.

Even though TV-DDM wins for both types of stimuli, the parameter estimates revealed quantitative differences between the two types of stimuli. The parameters show faster integration (β) and fewer stages (*n*) in the RDP task than in the RDK task. These findings are plausible as it makes sense that the processing of color involves fewer stages and is quicker as it does not involve the integration of perceptual information across time compared to information about motion. In fact, the integration time is around 100 ms faster. This difference is broadly consistent with the well-known effect of color-motion asynchrony, which indicates that humans perceive color earlier than motion by around 80 ms (e.g., [64,65]; see [71] for DDM-based approach and a comprehensive review). It is also interesting to note that the integration time of around 300 ms is similar to Smith and Lilburn’s [19] finding of 400 ms (which, in turn, is consistent with Watamaniuk and Sekuler’s [18] findings). The shorter duration may be because our RDK’s were smaller and contained fewer dots, i.e., less perceptual information to integrate. Moreover, even though the distributional measures indicate a good fit, particularly for short RTs, a closer inspection for intermediate and longer RTs suggests that the exact shape of the gamma function may not precisely describe the perceptual integration. Study 1 suggests that for high noise, RDK integration proceeds slightly slower compared to low noise stimuli. For RDPs, the integration may be slightly faster for longer RTs. These mismatches may not be surprising as the neural processing of perceptual stimuli is highly non-linear (see [9] for evidence pointing in this direction), inconsistent with linear filtering foundations of the gamma function. In fact, Smith and Lilburn [19] did acknowledge such potential problems with the design of the gamma function. Nevertheless, the gamma function may be a good compromise between a highly complex function and the oversimplification of the formation of perceptual representation in the DDM.

Finally, we found a violation of selective influence for the response boundary in both models and both tasks. This finding suggests that the change in drift rate across noise levels is not sufficient to capture the change in the RT distribution’s shape across noise levels, e.g., the leading edge, and therefore mandates additional changes in the response boundary. The fact that this effect is also found in DDM suggests that this is not due to the additional perceptual integration but is an effect on the decision-making stage. Importantly, these findings point to within-trial changes in response boundary, as the estimates indicate that participants adapt the response boundary to each noise level despite this factor being randomized across trials, which prevents them from adapting the response boundary to each noise level at the beginning of a trial. However, in contrast to the common finding of “collapsing” boundaries (e.g., [41,42,43]) being associated with higher costs or response deadlines, in our case, the increase in task difficulty leads to an increase in response boundary rather than a decrease. Interestingly, this is consistent with findings by Lin et al. [6] in a visual search study where participants were tasked with finding a designated target among non-targets. They found that the response boundary increased with an increasing number of objects on the screen (i.e., the difficulty of the search task). Also, the theoretical study by Malhotra et al. [44] showed that a certain mix of easy and difficult decisions can also lead to an increase in the response boundary. This particular effect could be found if blocked easy conditions result in a lower response boundary than blocked difficult conditions. In this scenario, the study showed that initial low boundaries are raised when the critical amount of evidence for the easy condition cannot be obtained very quickly, and it is getting increasingly evident that it is a difficult stimulus i.e., requiring a higher respond boundary to initiate a correct decision. Future studies will have to examine this prediction and examine the conditions in our design individually. Moreover, future studies should fit formal models of collapsing boundaries to our data (e.g., [41,42,43,72]). Another alternative to explaining our findings are urgency-gating models (e.g., urgency models [73,74]) using our using our Bayesian parameter estimation and model comparison.

## Figures and Tables

**Table 1:** Prior distributions used in both the motion and color discrimination tasks. TN represents the probability density function for a truncated normal distribution in the form TN(µ, σ, lower truncation, upper truncation).

DDM	TV-DDM
A~ TN(0.17, 0.17, 0, ∞)	A~ TN(0.2, 0.2, 0, ∞)
Ter~ TN(0.26, 0.26, 0, ∞)	Ter~ TN(0.18, 0.18, 0, ∞)
v~ TN(0.25, 0.25, 0, ∞)	v~ TN(0.3, 0.3, 0, ∞)
sz~ TN(0.05, 0.05, 0, ∞)	$\sigma^{2}\sim \;TN(0.1,\;0.1,\;0,\;\infty )$
sv~ TN(0.07, 0.07, 0, ∞)	sv~ TN(0.25, 0.25, 0, ∞)
ster~ TN(0.28, 0.28, 0, ∞)	β~ TN(25, 25, 0, ∞)
	n~ TN(5, 5, 0, ∞)

**Figure A1:**
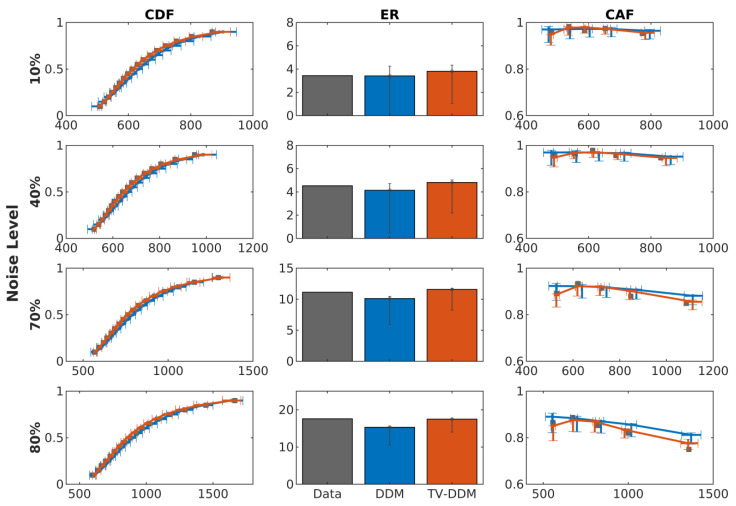
Cumulative density functions (CDF), average error rate (ER), and conditional accuracy functions (CAF) for the data and both models averaged over participants in the RDK task. The error bar shows the 95% credible interval, indicating the variability in the distributional measures due to the uncertainty of the parameter estimates (posterior distribution).

**Figure A2:**
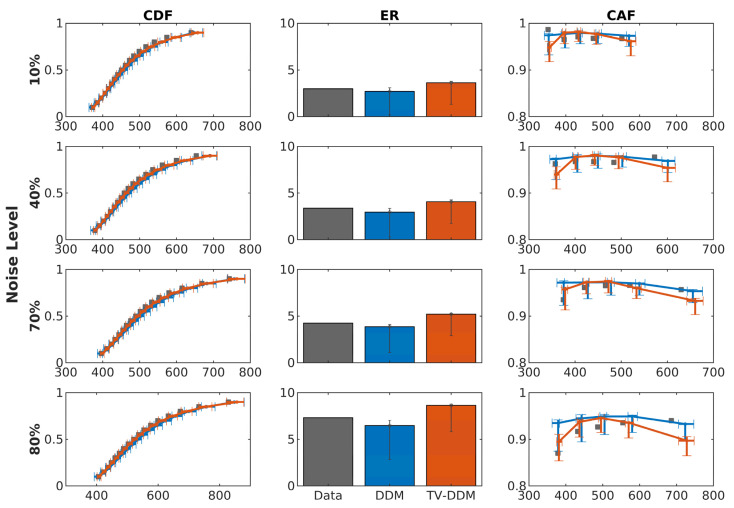
Cumulative density functions (CDF), average error rate (ER), and conditional accuracy functions (CAF) for the data and both models averaged over participants in the RDP task. The error bar shows the 95% credible interval, indicating the variability in the distributional measures due to the uncertainty of the parameter estimates (posterior distribution).

## Data Availability

The raw data supporting the conclusions of this article will be made available by the authors on request.

## Appendix A

**Table A1:** Results of the one-way ANOVA on the EAP estimates from the motion discrimination task for the TV-DDM.

Predictor	*Df*	*F*	*p*	$\eta_{p}^{2}$	Posterior Overlap
*A*					
Noise	3	44.93	<0.001	0.500	0
*Ter*					
Noise	3	1.14	0.335	0.025	0.98
*V*					
Noise	3	153.59	<0.001	0.773	0
*σ* ^2^					
Noise	3	47.56	<0.001	0.433	0.00
*beta*					
Noise	3	10.57	<0.001	0.190	0.15
*n*					
Noise	3	4.98	0.003	0.100	0.62
*Sv*					
Noise	3	7.800	<0.001	0.148	0.0
Error (Noise)	135				

**Table A2:** Results of one-way ANOVA on the EAP estimates from the color discrimination task for the TV-DDM.

Predictor	*Df*	*F*	*p*	$\eta_{p}^{2}$	Posterior Overlap
*A*					
Noise	3	12.48	<0.001	0.266	0.00
*Ter*					
Noise	3	1.50	0.219	0.041	0.97
*v*					
Noise	3	7.09	<0.001	0.168	0.029
*σ* ^2 ^					
Noise	3	22.00	<0.001	0.386	0.0
*β*					
Noise	3	29.88	0.340	0.031	0.95
*n*					
Noise	3	0.212	0.888	0.006	1.00
*sv*					
Noise	3	1.91	0.133	0.052	0.94
Error (Noise)	105				

**Table A3:** Results of the one-way ANOVA on the EAP estimates from the motion discrimination task for the DDM.

Predictor	*df*	*F*	*p*	$\eta_{p}^{2}$	Posterior Overlap
*A*					
Noise	3	13.68	<0.001	0.233	0
*Ter*					
Noise	3	3.84	0.011	0.079	0.24
*V*					
Noise	3	193.80	<0.001	0.811	0
*sz*					
Noise	3	1.53	0.210	0.033	0.93
*Ster*					
Noise	3	37.95	<0.001	0.458	0
*Sv*					
Noise	3	0.79	0.50	0.017	0.995
Error (Noise)	135				

**Table A4:** Results of the one-way ANOVA on the EAP estimates from the color discrimination task for the DDM.

Predictor	*Df*	*F*	*p*	$\eta_{p}^{2}$	Posterior Overlap
*A*					
Noise	3	2.90	0.039	0.076	0.51
*Ter*					
Noise	3	2.52	0.06	0.067	0.72
*V*					
Noise	3	20.49	<0.001	0.368	0
*sz*					
Noise	3	10.21	<0.001	0.226	0.038
*Ster*					
Noise	3	4.24	0.007	0.108	0.33
*Sv*					
Noise	3	0.70	0.55	0.020	1
Error (Noise)	135				

**Table A5:** Results of the 2 (task) × 4 (noise) ANOVA on the EAP estimates from the color and motion discrimination tasks for the TV-DDM.

TV-DDM				
Predictor	*df*	*F*	*p*	$\eta_{p}^{2}$
*A*				
Task	1	14.274	<0.001	0.151
Noise	3	51.019	<0.001	0.389
Task × Noise	3	4.999	0.002	0.059
*Ter*				
Task	1	52.661	<0.001	0.397
Noise	3	1.135	0.336	0.014
Task × Noise	3	1.323	0.267	0.016
*V*				
Task	1	78.410	<0.001	0.495
Noise	3	87.420	<0.001	0.522
Task × Noise	3	25.631	<0.001	0.243
*σ^2^*				
Task	1	7.424	0.008	0.085
Noise	3	61.141	<0.001	0.433
Task × Noise	3	4.801	0.003	0.057
*Β*				
Task	1	120.070	<0.001	0.600
Noise	3	1.587	0.193	0.019
Task × Noise	3	7.554	<0.001	0.086
*N*				
Task	1	30.382	<0.001	0.275
Noise	3	2.144	0.095	0.026
Task × Noise	3	1.248	0.293	0.015
*Sv*				
Task	1	3.626	0.060	0.043
Noise	3	7.832	<0.001	0.089
Task × Noise	3	0.405	0.749	0.005
Error (Task)	80			
Error (Noise)	240			

**Table A6:** Results of the 2 (task) × 4 (noise) ANOVA on the EAP estimates from the color and motion discrimination tasks for the DDM.

DDM				
Predictor	*df*	*F*	*p*	$\eta_{p}^{2}$
*A*				
Task	1	6.630	0.012	0.077
Noise	3	13.540	<0.001	0.145
Task × Noise	3	1.688	0.170	0.021
*Ter*				
Task	1	101.824	<0.001	0.560
Noise	3	4.909	0.002	0.058
Task × Noise	3	1.161	0.325	0.014
*V*				
Task	1	56.986	<0.001	0.961
Noise	3	138.671	<0.001	0.634
Task × Noise	3	18.949	<0.001	0.192
*Sz*				
Task	1	14.899	<0.001	0.157
Noise	3	11.678	<0.001	0.127
Task × Noise	3	6.119	<0.001	0.071
*ster*				
Task	1	140.519	<0.001	0.637
Noise	3	33.558	<0.001	0.296
Task × Noise	3	15.221	<0.001	0.169
*Sv*				
Task	1	18.191	<0.001	0.185
Noise	3	0.897	0.444	0.011
Task × Noise	3	0.670	0.571	0.008
Error (Task)	80			
Error (Noise)	240			
